# Pneumocystis pneumonia occurrence and prophylaxis duration in kidney transplant recipients according to perioperative treatment with rituximab

**DOI:** 10.1186/s12882-020-01750-8

**Published:** 2020-03-11

**Authors:** Young Hoon Kim, Jee Yeon Kim, Dong Hyun Kim, Youngmin Ko, Ji Yoon Choi, Sung Shin, Joo Hee Jung, Su-Kil Park, Sung-Han Kim, Hyunwook Kwon, Duck Jong Han

**Affiliations:** 1 Division of Kidney and Pancreas Transplantation, Department of Surgery, Asan Medical Center, University of Ulsan College of Medicine, 88, Olympic-ro 43-gil, Songpa-gu, Seoul, 05505 South Korea; 2 Division of Nephrology, Department of Internal Medicine, Asan Medical Center, University of Ulsan College of Medicine, Seoul, South Korea; 3 Department of Infectious Diseases, Asan Medical Center, University of Ulsan College of Medicine, Seoul, South Korea

**Keywords:** Kidney transplantation, Rituximab, Pneumocystis

## Abstract

**Background:**

Pneumocystis pneumonia (PCP) is a life-threatening fungal infection that can occur in kidney transplantation (KT) recipients. A growing number of KT recipients are receiving perioperative treatment with rituximab, which is associated with prolonged B-cell depletion and possible risk of PCP occurrence; however, the optimal prophylaxis duration according to rituximab treatment is yet unknown. We compared the occurrence of PCP and the duration of prophylaxis in KT recipients according to rituximab treatment.

**Methods:**

We retrospectively analyzed 2110 patients who underwent KT between January 2009 and December 2016, who were divided into non-Rituximab group (*n* = 1588, 75.3%) and rituximab group (*n* = 522, 24.7%).

**Results:**

In the rituximab group, the estimated number needed to treat (NNT) for prophylaxis prolongation from 6 to 12 months was 29.0 with a relative risk reduction of 90.0%. In the non-rituximab group, the estimated NNT value was 133.3 and the relative risk reduction was 66.4%. Rituximab treatment (hazard ratio (HR) = 3.09; *P* <  0.01) and acute rejection (HR = 2.19; *P* = 0.03) were significant risk factors for PCP in multivariate analysis.

**Conclusions:**

Our results suggest that maintaining PCP prophylaxis for 12 months may be beneficial in KT recipients treated with rituximab for desensitization or acute rejection treatment.

## Background

Pneumocystis pneumonia (PCP), which is caused by Pneumocystis jiroveci (*P. jiroveci*), is a life-threatening fungal infection that can occur in renal transplant recipients [1]. Following the implementation of PCP prophylaxis using trimethoprim–sulfamethoxazole (TMP–SMX), the incidence of PCP in kidney transplantation (KT) recipients decreased from approximately 10 to 1% [2, 3]. Most cases of PCP occur several months after prophylaxis discontinuation [3, 4]; yet, current recommendations for duration of prophylaxis vary widely from 3 to 12 months [5, 6]. Studies have shown that factors such as age, cytomegalovirus (CMV) infection, lymphopenia, immunosuppressive regimen, and acute graft rejection may serve as indications for extended prophylaxis; however, the exact duration of prophylaxis needed in each patient is not established, especially in KT recipients who had been treated with rituximab, a monoclonal antibody against CD20, for pre-transplant desensitization or rejection treatment after transplant [2, 5, 7, 8].

Rituximab, which has a long-lasting effect in depleting B-cells for 6–12 months, is a good option for pre-transplant and adjuvant treatment [9, 10]. However, recent studies have reported that rituximab increases the risk of opportunistic infections including PCP with fulminant clinical course and mortality [11–13]. Since 2009, approximately 20% of KT performed at our center undergo pre-transplant desensitization with rituximab in order to overcome the human leukocyte antigen (HLA) or blood group A/B barriers [11, 14]. After encountering rituximab-treated KT recipients who later developed PCP at few months after prophylaxis discontinuation, we sought to obtain empirical evidence for the benefit of prolonged prophylaxis duration. We assessed the optimal duration of prophylaxis for PCP following KT in recipients who were treated with rituximab for pre-transplant desensitization or rejection treatment within 6 months after transplant.

## Methods

### Patients

This was a single-center, retrospective study using data extracted from the registry of Asan Medical Center (AMC) in Seoul, Korea; the data base was manually renewed database by reviewing the medical records of patients who underwent KT (AMC IRB number 2018–0207). The institutional review board at AMC approved the protocols of this study. We included consecutive patients who underwent KT at our center between January 2009 and December 2016; we excluded 24 patients who did not maintain TMP–SMX prophylaxis due to side effects (17 patients) and noncompliance (7 patients) and 2 patients who were treated with rituximab due to malignant disease after KT. The study cohort was divided into non-rituximab group and rituximab group, the latter of which was defined as recipients who had been treated with rituximab due to pre-operative desensitization or rejection treatment within 6 months after transplant. The patients who underwent rejection treatment using rituximab in the 6 months after KT were not included in the rituximab group.

### PCP prophylaxis and diagnosis

The PCP-prophylaxis protocol consisted of TMP–SMX (80–400 mg) daily for all recipients during the first 6 months following KT. We also administered additional 6 months of TMP–SMX prophylaxis to recipients who received any kind of immunologic treatment due to rejection. Diagnosis of PCP was confirmed through immunofluorescence or immunohistochemical antibody assay in bronchoalveolar lavage fluids in patients who had suspicious symptoms or radiologic findings.

### Desensitization and immunosuppression

According to our desensitization protocols, a single dose of rituximab (200–500 mg) was administered 1–2 weeks prior to plasmapheresis with or without intravenous immunoglobulin in both ABO-incompatible (ABOi) and crossmatch (XM)-positive KT recipients [11]. XM-positive KT was defined as complement-dependent cytotoxicity or flow-cytometric (FC) XM-positive KT. T-cell and B-cell FCXM-positive cases were defined by the ratio of median fluorescence intensity to control median values exceeding 2.0 and 2.5 (77 for T-cell and 101 for B-cell FCXM for a median channel shift on a 1024 scale), respectively. For induction, basiliximab (anti-IL-2 receptor antibody) or anti-thymocyte globulin (ATG) was used; for maintenance, calcineurin inhibitor (target trough level: tacrolimus [5–7 ng/ml], cyclospirin [100–150 ng/mL]), corticosteroid, and mycophenolic acid were used.

### Definition

CMV infection was monitored using CMV PCR or CMV antigenemia in blood samples at 1, 2, 3, 4, 6, 8, 12, 16, 20, and 24 weeks after KT and annually thereafter. In recipients with CMV viremia, pre-emptive treatment was performed rather than routine prophylaxis. We did not perform the protocol biopsy during the study period. Renal biopsy was conducted in patients suspicious of acute rejection, and the diagnosis was made pathologically in accordance with the Banff criteria [15]. The number needed to treat (NNT) is the number of patients who would need to be treated to prevent one adverse event; this metric has become useful for interpreting treatment benefits [16, 17]. It is calculated as the reciprocal of the absolute risk reduction between two study groups [16].

### Statistics

Categorical variables were compared by the Chi-squared test or Fisher’s exact test, as appropriate and continuous variables were compared with the Student’s *t-*test. The incidence rate of PCP was evaluated with the Kaplan–Meier method and compared between the two groups with the log-rank test. The risk factors for occurrence of PCP after KT were evaluated using univariate and multivariate Cox proportional hazard regression analysis. Risk factors of PCP related to indications for rituximab treatment (XM-positive, ABOi, and rejection treatment within 6 months after transplant) were analysed separately using Cox proportional hazards regression analysis because these factors are considered subgroups of the rituximab group, using the non-rituximab group as a reference. The NNT was applied to evaluate the benefit of prolonging prophylaxis duration from 6 months to 12 months. Considering the fact that the current study is not a case-control study, we introduced the concept of estimated NNT based on the assumption that no PCP occurred during TMP–SMX prophylaxis. *P-*values < 0.05 were considered statistically significant, and all statistical analyses were performed with SPSS version 18.0 (SPSS Inc., Chicago, IL, USA).

## Results

### Patient demographic and clinical characteristics

**Fig. 1:**
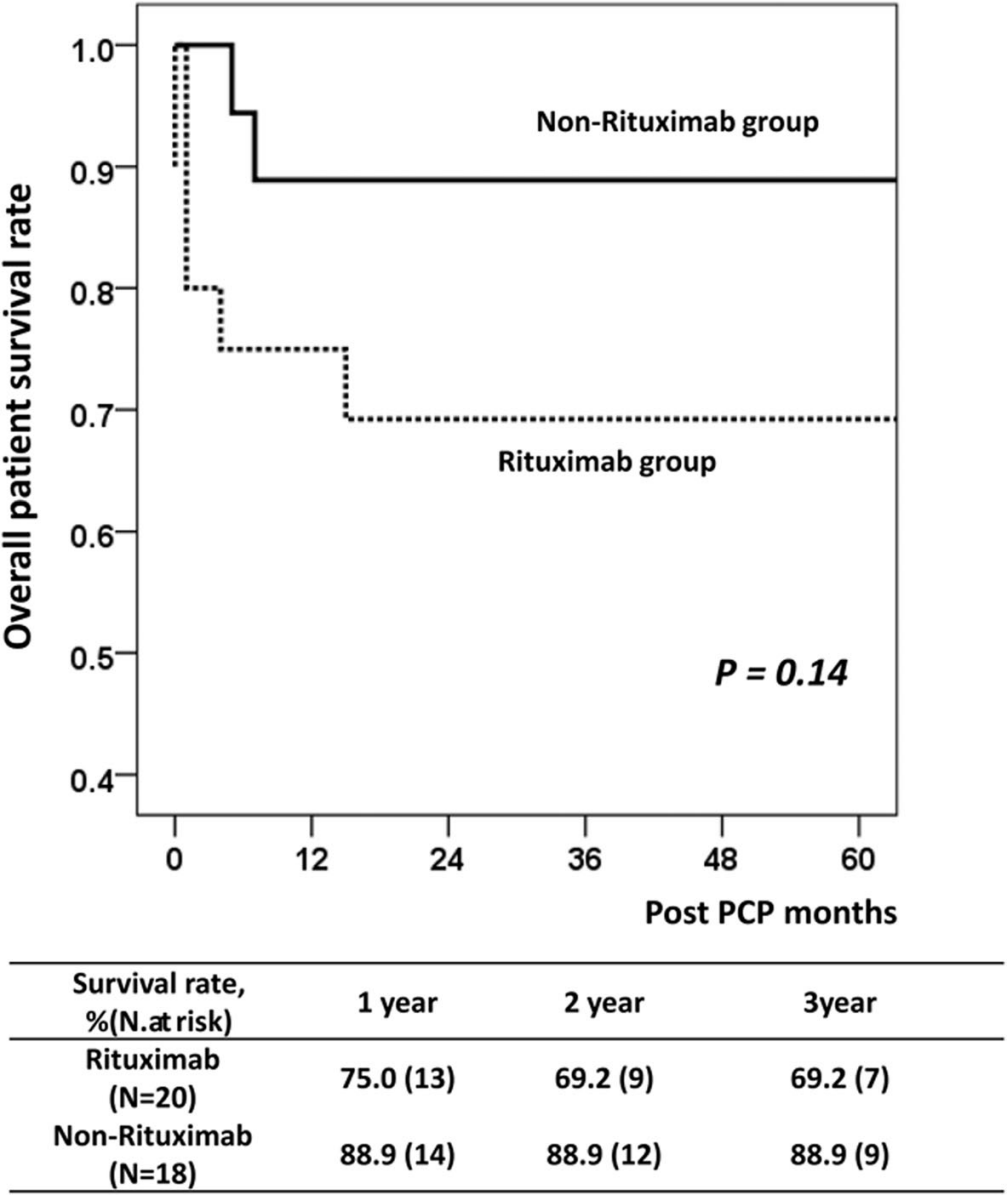
Overall patient survival after *Pneumocystis jiroveci* pneumonia

Out of the 2119 consecutive patients who received KT at our center, we excluded 7 patients who did not maintain TMP–SMX prophylaxis and 2 patients who received rituximab for malignant disease after KT. Finally, 2110 patients were included in the analysis, who were divided into the non-rituximab group (*n* = 1588, 75.3%) and the rituximab group (*n* = 522, 24.7%). The baseline and clinical characteristics of the study population are shown in Table 1**.** The rituximab group had significantly higher proportion of females (*P <* 0.01) and living donors (*P <* 0.01), history of previous KT (*P <* 0.01), higher degree of HLA class I and II PRA (*P <* 0.01), and higher prevalence of CMV viremia following KT (*P <* 0.01). Rituximab treatment was administered for desensitization owing to XM positivity in 126 (24.1%) patients, ABO incompatibility in 331 (63.4%) patients, both XM positivity and ABO incompatibility in 51 (9.8%) patients, and treatment of rejection that occurred within 6 months after KT in 14 (2.7%) patients. PCP occurred more frequently in the rituximab group (*n* = 20, 3.8%) than in the non-rituximab group (*n* = 18, 1.1%) (*P <* 0.01). The two groups did not show significant differences in terms of age, body mass index, and prevalence of diabetes mellitus and hypertension. Overall, PCP-related mortality rate was significantly higher in the rituximab group (*n* = 5, 1.0%) than non-Rituximab group (*n* = 1, 0.1%) (*P <* 0.01). Among the 38 patients who were diagnosed as PCP, the rituximab group had a tendency toward higher mortality rate (5/20, 25.0%) compared with the non-rituximab group (1/18, 5.6%), albeit without statistical significance (*P =* 0.18) (Fig. 1).

**Table 1:** Baseline and clinical characteristics of study patients

	Non-rituximab group	Rituximab group	*P*-value
Number of patients	1588 (75.3)	522 (24.7)	
Mean age (years)	47.1 ± 11.2	47.6 ± 11.5	0.37
Female sex	610 (39.3)	275 (49.3)	< 0.01
Diabetes mellitus	325 (20.5)	114 (21.8)	0.51
Hypertension	1376 (86.6)	459 (87.9)	0.50
Body mass index (kg/m^2^)	24.4 ± 6.5	23.0 ± 7.8	0.64
Cause of rituximab treatment			
XM positive	–	126 (24.1)	
ABO- i	–	331 (63.4)	
XM positive & ABO- i	–	51 (9.8)	
Rejection treatment	–	14 (2.7)	
Calcineurin inhibitor			0.66
Prograf	1165 (73.4)	388 (74.3)	
Cyclosporin	423 (26.6)	134 (25.7)	
Induction			< 0.01
No induction	81 (5.1)	0 (0.0)	
ATG	121 (7.6)	3 (0.6)	
Basiliximab	1386 (87.3)	519 (99.4)	
Previous transplant	114 (7.2)	51 (9.8)	< 0.01
Duration of dialysis (months)	41.8 ± 54.3	23.4 ± 35.7	< 0.01
Donor			< 0.01
Deceased donor	439 (27.6)	4 (0.8)	
Living related	761 (47.9)	275 (52.7)	
Living unrelated	388 (24.5)	243 (46.5)	
HLA-A,B,DR mismatch	3.0 ± 1.7	3.4 ± 1.7	< 0.01
PRA class I	10.3 ± 22.7	18.0 ± 30.8	< 0.01
PRA class II	10.9 ± 24.0	18.9 ± 32.4	< 0.01
CMV viremia	393 (35.7)	223 (43.1)	< 0.01
PCP after transplant	18 (1.1)	20 (3.8)	< 0.01
PCP related mortality	1 (0.1%)	5 (1.0%)	< 0.01
Mortality among PCP patients (*n* = 38)	1 (5.6%)	5 (25.0%)	0.18

Continuous data are presented as means ± standard deviations, and categorical data are presented as number (%)

XM, crossmatching; *ABO-i* ABO incompatible, *ATG* anti-thymocyte globulin, *HLA* human leukocyte antigen, *PRA* panel reactive antibody, *CMV* Cytomegalovirus, *PCP* pneumocystis carinii pneumonia

### Number needed to treat

**Fig. 2:**
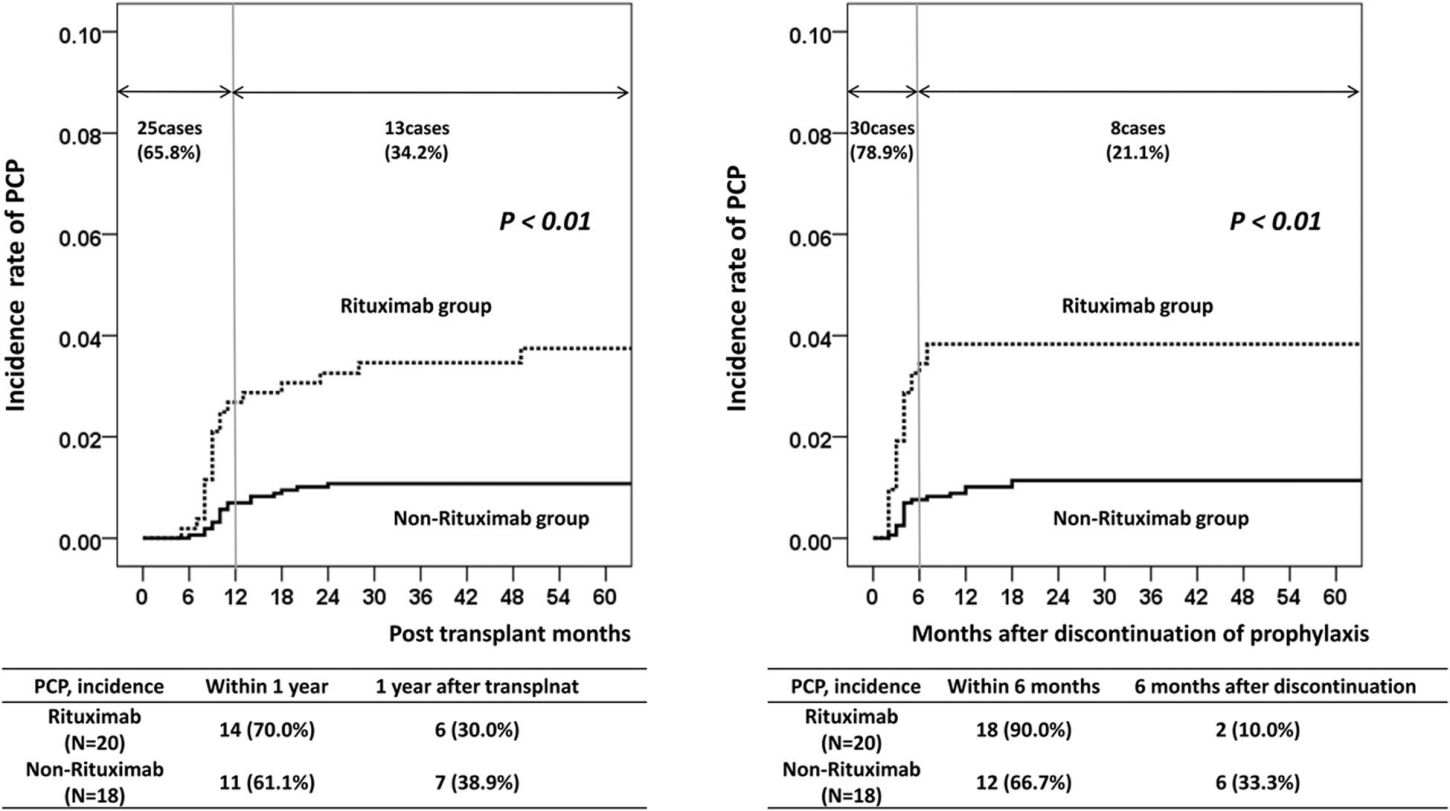
Incidence of *Pneumocystis jiroveci* pneumonia after (**a**) transplant and (**b**) discontinuation of prophylaxis

The majority of PCP cases were diagnosed within 1 year after transplant (25/38, 64.8%) and within 6 months after discontinuation of prophylaxis (30/38, 78.9%). In the rituximab group, 18 (90.0%) out of the 20 PCP cases occurred within 6 months after the discontinuation of TMP–SMX prophylaxis (Fig. 2).

The estimated NNT according to TMP–SMX prophylaxis duration are shown in Table 2**.** In the rituximab group, the estimated NNT for prophylaxis prolongation from 6 to 12 months was 29.0 to prevent 1 case of PCP with 90.0% of relative risk reduction; among the total 20 cases of PCP in the rituximab group, 18 cases (90.0%) would have been preventable if 12 months of prophylaxis were implemented. In the non-rituximab group, the estimated NTT value was 133.3 and the relative risk reduction was 66.4%.

**Table 2:** Estimated number needed to treat according to Trimethoprim/sulfamethoxazole prophylaxis duration

	12-months prophylaxis	6-months prophylaxis
Rituximab group		
PCP events	2	20
Non-PCP events	520	502
Total subjects	522	522
PCP incidence	0.0038 (0.38%)	0.0383 (3.83%)
Absolute risk reduction	3.45%	
Relative risk reduction	90.0%	
Number needed to treat	29.0	
Non-rituximab group		
PCP events	6	18
Non-PCP events	1582	1570
Total subjects	1588	1588
PCP incidence	0.0038 (0.38%)	0.0113 (1.13%)
Absolute risk reduction	0.75%	
Relative risk reduction	66.4%	
Number needed to treat	133.3	

*PCP* pneumocystis carinii pneumonia

### Risk factors associated with PCP

In the univariate regression analysis, rituximab dose, sex, XM positivity, ABO incompatibility, and ATG had no significant association with PCP occurrence. Rituximab treatment (*P* <  0.01), acute rejection (P <  0.01), and CMV viremia (*P* = 0.04) were independent risk factors in the univariate analysis. After adjusting for confounding factors, rituximab treatment (hazard ratio (HR) = 3.09; 95% confidence interval [CI], 1.60–5.96; P <  0.01) and acute rejection PCP (HR = 2.19; 95% CI, 1.09–4.14; *P* = 0.03) remained as independent risk factors for PCP occurrence, but CMV viremia prior to PCP was not a significant risk factor in the multivariate Cox proportional hazard regression analysis (HR = 1.62; 95% CI, 0.82–3.20; *P* = 0.16). (Table 3) Additional univariate and multivariate regression analyses were conducted on patients in the rituximab group to evaluate risk factors of PCP according to rituximab treatment indication. ABO incompatibility (HR = 2.44; 95% CI, 1.09–5.48; *P* = 0.04) and rejection treatment using rituximab within 6 months after transplant (HR = 77.61; 95% CI, 26.35–228.60; *P* <  0.01) were found to be significant factors associated with PCP occurrence (Table 4).

**Table 3:** Factors associated with the occurrence of pneumocystis carinii pneumonia

	Univariate analysis		Multivariate analysis	
	HR (95% CI)	*P*-value	HR (95% CI)	*P*-value
Rituximab treatment	3.47 (1.84–6.56)	< 0.01	3.09 (1.60–5.96)	< 0.01
Rituximab dose = 0 mg	Reference			
≤ 200 mg	1.84 (0.89–3.82)	0.12	–	–
> 200 mg	0.77 (0.18–3.23)	0.72	–	–
Male vs. female	1.40 (0.74–2.64)	0.30	–	–
Acute rejection	2.46 (1.23–4.92)	0.01	2.19 (1.09–4.14)	0.03
ABO-compatible and XM-negative	Reference		–	–
ABO-incompatible	0.48 (0.07–3.56)	0.47	–	–
XM-positive	1.86 (0.89–3.89)	0.10	–	–
ABO-incompatible and XM-positive	1.18 (0.16–8.90)	0.87	–	–
ABO incompatibe vs. compatible	0.66 (0.16–2.75)	0.56	–	–
XM positive vs. negative	3.66 (0.33–40.37)	0.29		
ATG vs. basiliximab	0.85 (0.20–3.57)	0.85	–	–
CMV viremia prior to PCP	1.94 (1.00–3.75)	0.05	1.62 (0.82–3.20)	0.16

*ATG* anti-thymocyte globulin, *CMV* Cytomegalovirus, *XM* crossmatch, *PCP* pneumocystis carinii pneumonia

**Table 4:** Risk factors of pneumocystis carinii pneumonia according to rituximab treatment indication

	Univariate analysis		Multivariate analysis	
	HR (95% CI)	*P*-value	HR (95% CI)	*P*-value
Non-rituximab group	Reference		Reference	
XM-positive	0.69 (0.09–5.20)	0.72	0.63 (0.08–4.78)	0.66
ABO-incompatible	2.49 (1.12–5.54)	0.03	2.44 (1.09–5.48)	0.04
ABO- incompatible and XM- positive	1.67 (0.22–12.50)	0.62	1.53 (0.20–11.54)	0.68
Rejection treatment within 6 months after transplant	91.9 (41.10–205.69)	< 0.01	77.61 (26.35–228.60)	< 0.01

XM, crossmatch

## Discussion

By studying a total of 38 cases of PCP in 2110 KT recipients, we found that the majority of PCP cases occurred within 6 months after prophylaxis discontinuation, with 90.0% of patients who received rituximab experiencing PCP following discontinuation. The estimated NTT in the rituximab group was 29.0 in Rituximab group, whereas previous studies reported NTT values of 284 for aspirin in preventing cardiovascular events and 186 for statin in preventing myocardial infarction [18, 19]. Collectively speaking, our study provides a strong evidence for prolongation of prophylaxis duration to 12 months in KT recipients treated with rituximab during perioperative period to prevent fatal infectious complications such as PCP.

In our study, the rituximab group included recipients treated with rituximab due to rejection episodes within 6 months after transplant as well as those who received rituximab for pre-operative desensitization. We included such patients because 6 months is considered a critical period of PCP occurrence [6]; accordingly, additional treatment with rituximab within 6 months after KT was found to be a significant risk factor for PCP in our cohort. Among the 14 patients who received rituximab for rejection treatment within 6 months following KT, PCP occurred in 9 patients (64.3%) during few months after the discontinuation of TMP–SMX prophylaxis. Rejection within 6 months was significantly associated with PCP development in multivariate analysis, whether or not rituximab was used. Patients who underwent rejection treatment using rituximab had a 77.6-fold increased risk of PCP occurrence compared to the non-Rituximab group. Therefore, we strongly recommend that patients who undergo rejection treatment with rituximab should receive at least 6 months prophylaxis from the time of rejection treatment or 1 year from transplant. Similar to the results of recent studies, CMV viremia prior to PCP was a significant risk factor in univariate analysis but not in multivariate analysis [2, 7, 8]; this result may be due to the higher rate of CMV viremia in the rituximab group. Thus, our results indicate that CMV viremia can be a good marker for patients’ immune status but not for the risk of PCP. The additional analysis for risk factors of PCP occurrence in the rituximab group compared to the non-rituximab group identified ABO incompatibility as a significant risk factor. Although it was not a study related to PCP, Sharif et al. showed that ABOi patients were more likely to experience BKVN compared to HLAi recipients [20]. Further investigation is needed to evaluate the possibility that intrinsic attributes of ABO incompatibility contribute to an increased risk for infectious complications after KT.

A total of 7 patients in the non-rituximab group developed PCP 1 year after transplant: 6 patients experienced PCP within 14 to 24 months after KT, and 1 patient developed PCP at 91 months after KT. Goto et al. suggested administering lifelong prophylaxis to prevent PCP occurrence; however, PCP prophylaxis for more than 1 year may not be appropriate considering the NNT value of 133.3 for prophylaxis prolongation from 6 to 12 months in our study and the low overall incidence of PCP after 1 year post-transplant [21]. In the rituximab group, 6 patients developed PCP within 13 to 29 months after KT, all of whom had rejection treatment prior to PCP, including 4 patients who were treated with rituximab. Except for 2 patients who developed PCP at 7 months after discontinuation, 18 cases of PCP occurred within 6 months after prophylaxis discontinuation. Considering that PCP tended to occur within 1 year after KT in the rituximab group, we believe that with a proper duration of prophylaxis, PCP can be effectively prevented in patients who receive rituximab. Due to the limited number of patients, we could not analyze the effectiveness and necessity of 12-months prophylaxis after rituximab treatment for rejection treatment within 6 months after transplantation. Nevertheless, we suggest using 12-months prophylaxis for KT recipients who received rejection treatment, especially when they have other risk factors for PCP.

KT across the HLA and blood group A/B barriers has been recently increasing [11]. Various desensitization protocols have been developed for such immunologically high-risk groups, and rituximab is one of the main component of pre-conditioning strategies and rejection treatment [11, 14, 22]. However, PCP in KT recipients treated with rituximab have not been evaluated extensively. In solid organ transplantation, it is hard to determine the impact of a single risk factor in PCP risk due to the confounding effects of various immunno-suppressive regimens and comorbidities of recipients. In our retrospective study using the database of a single large center, we showed that perioperative rituximab treatment had 3.09 times higher hazards of PCP occurrence after adjusting for other risk factors, which is in line with the results of previous studies [2, 5, 7, 8]. Rituximab dose was suggested to be related with serious infectious complications following transplant, [11, 12] with Lee et al. reporting that recipients treated with standard dose rituximab had higher risk of fungal infection than those who received lower dose of rituximab [12]. In our results, rituximab dose did not show significant association with PCP occurrence; this result may be due to the abrupt and long-lasting effects of B-cell depression, and because the incidence of PCP was too small to obtain statistical significance [12].

CD4^+^ T lymphocytes orchestrate the defense against *P. jiroveci*, and low CD4+ T lymphocyte count is thus suggested as an independent risk factor associated with PCP in solid organ transplant recipients [2]. In vivo studies have suggested a mechanism for how rituximab may increase the risk of PCP by inducing B-cell depletion: [23, 24] Lind et al. showed that owing to the absence of *P. jiroveci*-specific antibody, mice with B-cell deficiency are more vulnerable to PCP, showing that as antigen presenting cells, B-cells play an important role in the defense response against *P. jiroveci* [23]. The same group also reported that B- and T-cell interaction carries a vital role in generating effector and memory CD4^+^ T lymphocyte response against *P. jiroveci* [24]. In addition, clinical studies on patients with hematologic malignancies supported the theory that B-cell suppression using rituximab increases the risk of PCP development [13, 25].

Recent studies showed that rituximab results in long-term elimination of B-cells up to more than 6 months, thereby suggesting prolongation of prophylaxis [9, 10]. Sidnet et al. reported that a single dose of rituximab in sensitized patients awaiting KT can induce rapid depletion of B-cell, which was maintained from 6 months to 1 year [9]. In addition, repopulation of functional B-cell subsets against microorganisms was predominantly preceded by CD19^+^CD5^+^ polyreactive B-cells and ontogenetically younger B-cells with reacting low affinity antibodies [9]. Ganberg et al. studied the effect of rituximab on B-cell populations in peripheral blood, within kidney biopsy tissues, and in inguinal lymph nodes in KT recipients who were maintained in conventional triple immunosuppressants; the authors showed that although the maximal effect was observed between 3 weeks to 6 months, B-cell populations remained suppressed up to several years [10]. In ABOi KT recipients, CD19^+^ cells did not recover after 12 months even after a single injection of reduced dose rituximab (200 mg) [12]. Our results further support the results of these studies and advocate the use of prolonged prophylaxis for 12 months.

This study is limited in that it was a retrospective study performed at a single center, which may have resulted in selection and information biases. Nevertheless, such study design also resulted in homogeneity of both study population and immunosuppressive protocol. Also, as most of the patients were of Asian descent, our results may have limited generalizability in other races. Lastly, basiliximab was primarily used as an induction treatment rather than ATG, especially in the rituximab group; although ATG was not a significant risk factor for PCP in our study, the incidence of PCP may be different in other clinical settings with different induction treatment protocols.

## Conclusions

We report that KT recipients who received rituximab for desensitization or treatment of acute rejection had higher incidence of PCP than those who did not receive rituximab, and that most cases of PCP (90.0%) occurred within 6 months following discontinuation of prophylaxis. Our results suggest that prolongation of PCP prophylaxis to 12 months may be beneficial in KT recipients who receive perioperative treatment with rituximab.

## Data Availability

The datasets analyzed during the current study are available from the corresponding author on reasonable request.

## Abbreviations

ABOi: ABO incompatible
ATG: anti-thymocyte globulin
CMV: cytomegalovirus
HLA: human leukocyte antigen
IF: immunofluorescence
KT: kidney transplantation
NNT: number needed to treatment
*P. jiroveci*: Pneumocystis jiroveci
PCP: Pneumocystis pneumonia
PCR: polymerase chain reaction
TMP–SMX: trimethoprim–sulfamethoxazole
XM: cross-match

## References

[1] Kinnunen S, Karhapaa P, Juutilainen A, Finne P, Helantera I (2018). Secular trends in infection-related mortality after kidney transplantation. Clin J Am Soc Nephrol.

[2] Iriart X, Challan Belval T, Fillaux J (2015). Risk factors of Pneumocystis pneumonia in solid organ recipients in the era of the common use of posttransplantation prophylaxis. Am J Transplant.

[3] Neofytos D, Hirzel C, Boely E, Lecompte T, Khanna N. *Pneumocystis jirovecii* pneumonia in solid organ transplant recipients: a descriptive analysis for the Swiss Transplant Cohort. Transpl Infect Dis. 2018;20(6):e12984. 10.1111/tid.12984 30155950

[4] Borstnar S, Lindic J, Tomazic J (2013). Pneumocystis jirovecii pneumonia in renal transplant recipients: a national center experience. Transplant Proc.

[5] Neff RT, Jindal RM, Yoo DY, Hurst FP, Agodoa LY, Abbott KC (2009). Analysis of USRDS: incidence and risk factors for Pneumocystis jiroveci pneumonia. Transplantation.

[6] Kidney Disease: Improving Global Outcomes (KDIGO) Transplant Work Group. KDIGO clinical practice guideline for the care of kidney transplant recipients. Am J Transplant. 2009;9(Suppl 3):S1–155. 10.1111/j.1600-6143.2009.02834.x 19845597

[7] Faure E, Lionet A, Kipnis E, Noel C, Hazzan M. Risk factors for Pneumocystis pneumonia after the first 6 months following renal transplantation. Transpl Infect Dis. 2017;19. 10.1111/tid.12735 28608641

[8] Lee SH, Huh KH, Joo DJ. Risk factors for *Pneumocystis jirovecii* pneumonia (PJP) in kidney transplantation recipients. Sci Rep. 2017;7:1571. 10.1038/s41598-017-01818-w PMC5431538 28484270

[9] Sidner RA, Book BK, Agarwal A, Bearden CM, Vieira CA, Pescovitz MD (2004). In vivo human B-cell subset recovery after in vivo depletion with rituximab, anti-human CD20 monoclonal antibody. Hum Antibodies.

[10] Genberg H, Hansson A, Wernerson A, Wennberg L, Tyden G (2007). Pharmacodynamics of rituximab in kidney transplantation. Transplantation.

[11] Kwon H, Kim YH, Choi JY (2016). Analysis of 4000 kidney transplantations in a single center: across immunological barriers. Medicine (Baltimore).

[12] Lee J, Lee JG, Kim S (2016). The effect of rituximab dose on infectious complications in ABO-incompatible kidney transplantation. Nephrol Dial Transplant.

[13] Martin-Garrido I, Carmona EM, Specks U, Limper AH (2013). Pneumocystis pneumonia in patients treated with rituximab. Chest.

[14] Kwon H, Kim YH, Choi JY, et al. Impact of pretransplant donor-specific antibodies on kidney allograft recipients with negative flow cytometry cross-matches. Clin Transplant. 2018;32:e13266. 10.1111/ctr.13266 29676812

[15] Haas M, Sis B, Racusen LC (2014). Banff 2013 meeting report: inclusion of c4d-negative antibody-mediated rejection and antibody-associated arterial lesions. Am J Transplant.

[16] Chong CA, Tomlinson G, Chodirker L (2006). An unadjusted NNT was a moderately good predictor of health benefit. J Clin Epidemiol.

[17] Laupacis A, Sackett DL, Roberts RS (1988). An assessment of clinically useful measures of the consequences of treatment. N Engl J Med.

[18] Xie M, Shan Z, Zhang Y (2014). Aspirin for primary prevention of cardiovascular events: meta-analysis of randomized controlled trials and subgroup analysis by sex and diabetes status. PLoS One.

[19] Sever PS, Poulter NR, Dahlof B, Wedel H (2005). Different time course for prevention of coronary and stroke events by atorvastatin in the Anglo-Scandinavian Cardiac Outcomes Trial-Lipid-Lowering Arm (ASCOT-LLA). Am J Cardiol.

[20] Sharif A, Alachkar N, Bagnasco S (2012). Incidence and outcomes of BK virus allograft nephropathy among ABO- and HLA-incompatible kidney transplant recipients. Clin J Am Soc Nephrol.

[21] Goto N, Takahashi-Nakazato A, Futamura K (2017). Lifelong prophylaxis with trimethoprim-Sulfamethoxazole for prevention of outbreak of Pneumocystis jirovecii pneumonia in kidney transplant recipients. Transplant Direct.

[22] Stegall MD, Gloor J, Winters JL, Moore SB, Degoey S (2006). A comparison of plasmapheresis versus high-dose IVIG desensitization in renal allograft recipients with high levels of donor specific alloantibody. Am J Transplant.

[23] Lund FE, Schuer K, Hollifield M, Randall TD, Garvy BA (2003). Clearance of Pneumocystis carinii in mice is dependent on B cells but not on P carinii-specific antibody. J Immunol.

[24] Lund FE, Hollifield M, Schuer K, Lines JL, Randall TD, Garvy BA (2006). B cells are required for generation of protective effector and memory CD4 cells in response to Pneumocystis lung infection. J Immunol.

[25] Jiang X, Mei X, Feng D, Wang X (2015). Prophylaxis and treatment of Pneumocystis jiroveci pneumonia in lymphoma patients subjected to rituximab-contained therapy: a systemic review and meta-analysis. PLoS One.

